# A dynamic block reward approach to improve the performance of blockchain systems

**DOI:** 10.7717/peerj-cs.1210

**Published:** 2023-01-13

**Authors:** Maher Alharby

**Affiliations:** Department of Computer Science, College of Computer Science and Engineering, Taibah University, Medina, Saudi Arabia

**Keywords:** Blockchain, Ethereum, Incentive, Performance, Throughput, Simulation, Security

## Abstract

In Ethereum, miners are responsible for expanding the blockchain ledger by appending new blocks of transactions in exchange for incentives. Within the current Ethereum incentive mechanism, miners can still receive a significant amount of reward when creating non-full or even empty blocks, despite their negative impact on the system performance. We provide an extensive data-driven analysis of the impact of non-full blocks on the system performance, with the help of the BlockSim simulation tool. We collect the data for 500,000 Ethereum blocks and fit the appropriate probability distributions to the data to provide input suitable for the simulator. We show that the performance of Ethereum can be improved by over 50% if all blocks were filled with transactions. We propose an adjustment to the current Ethereum incentive model to assure the received incentive is always proportional to the block utilization level. Using our proposed approach, the incentive for non-full blocks is significantly reduced, making this behavior less attractive for miners. This implies that miners would be enforced to fill their blocks with transactions, and thus the performance is pushed to its optimal level. We show that our approach can work in practice without any crucial security issues.

## Introduction

In blockchain systems, it is essential to ensure that the performance is pushed to its optimal level to speed up the processing time for transactions. One simple approach to improve the performance of blockchain systems is through increasing the size of blocks to handle more transactions. However, as blockchains rely on miners to maintain the blockchain ledger, the behavior of miners can also play a significant role in the system’s performance. For example, when miners do not fill their blocks with transactions this can negatively impact the performance level.

Public blockchains such as Ethereum have built-in incentive mechanisms to provide incentives for miners to operate and maintain the blockchain correctly. Such incentive mechanisms should be enough to assure that miners would maintain the blockchain securely and properly. For instance, the incentive mechanism should enforce miners to push the system performance to its optimal level by filling their blocks with transactions.

In this article, we conduct the first analysis of its kind to assess the impact of producing non-full blocks on the performance of the Ethereum system. Our aim is not only to understand the reasons behind such behavior but also to propose a solution that assures the proper utilization of the created blocks, thus pushing the system performance to the optimal level. We follow a model-based approach to carry out our analysis of different configurations and scenarios since it is not feasible or practical to get insights about the implications of non-utilized blocks only based on observation of the real Ethereum network. That is, a model-based approach augmented with data-driven parameterization stands as the only reasonable approach.

Different techniques have been utilized to achieve our analysis in this work. Firstly, we make use of the BlockSim simulation tool. This simulator provides generic simulation constructs that can be extended to study the performance of any blockchain system of interest (Alharby & van Moorsel, 2019). We extend some of the simulator’s functionalities to suit our analysis. Secondly, to conduct data-driven simulation studies, we collect the data for about 500 thousand Ethereum blocks from the real Ethereum network. This is to parameterize the simulator with real data. Thirdly, we utilize statistical techniques to transform the collected data into distributions to parameterize the simulator. For instance, we use Gaussian Mixture Models to fit distributions to the Used Gas data for transactions.

The main insights of our analysis are as follows. Over the past years, the Ethereum foundation has increased the block limit to handle more transactions, and thus improve the system performance. However, we found that this solution does not guarantee the best achievable performance level. The performance of Ethereum can be improved by over 50% if blocks were filled up with transactions. For example, the number of transactions processed per second in the current implementation of Ethereum can be pushed from 14 to 22 if all blocks are filled with transactions. This indicates that the performance level does not solely depend on the block limit, but it also depends on other factors such as the behavior of miners and the built-in incentive mechanism.

Our analysis also suggests that the current Ethereum incentive model is not properly designed as it leaves advantages for miners to produce non-full or even empty blocks, which in turn reduces the system performance level regardless of the block limit. To address this, we propose an adjustment to the Ethereum incentive model to significantly reduce the amount of rewards miners would get for producing non-full blocks. The intention behind our proposed approach is to enforce miners to utilize their blocks properly, and thus the system performance will be pushed to its optimal level. Our approach does not guarantee that miners would fill their blocks with transactions as this depends on their behaviors, but it assures that miners will not benefit from creating such non-full blocks. Using our proposed approach, miners who fill up to 20% of their blocks, for instance, can only get one-fifth of the rewards they are currently getting in Ethereum. This means our approach results in a significant reduction in the mining incentives for non-full blocks, making the behavior of creating non-full blocks less attractive for miners. The proposed scheme can work in practice and has no crucial security issues, as we will discuss in the “Discussion” section.

The structure of this article is as follows. “Background” provides an introduction to the topics relevant to the work presented in this article. “Proposed Dynamic Block Reward Approach” introduces the problem space of establishing non-full blocks and our proposed approach of dynamic block rewards to improve the system performance. We discuss the data collection and fitting exercise for Ethereum data in “Data Collection and Distribution Fitting”. This includes the details of the statistical techniques used to derive the right distributions. “Simulator and Validation of Simulation Results” describes how we used and extended the BlockSim simulator to suit our study. This section also provides a simulation experiment to validate the simulation results by comparing them with that of the real Ethereum system. “Results” provides the results of the simulation study, for different configurations as well as the results for our proposed approach. We discuss the validity threats to our analysis in addition to the adoption and security aspects of the proposed scheme in “Discussion”. Finally, we discuss the state-of-the-art literature in “Related Work” and conclude the article in “Conclusion”.

## Background

A blockchain is a distributed ledger that is replicated and shared between the network’s participants. This ledger is composed of linked blocks, each of which embraces a number of transactions. The main intention behind blockchain technology is that it eliminates the need for trust among the network’s participants. That is, non-trusting participants can securely communicate with each other even in the absence of intermediaries (Nakamoto, 2008).

The blockchain network is composed of several nodes, known as miners, who are responsible for expanding the blockchain ledger by attaching new blocks. To create and attach a new block to the ledger, miners first select several transactions to be executed and included in the block by engaging in a consensus mechanism such as Proof-of-Work (PoW). The created block is then propagated to other nodes in the network to update their local ledgers. The recipient nodes are expected to verify the block and its embedded transactions before adding it to their ledgers. Once the created block is accepted by the nodes and it is appended to the global ledger, the miner of that block will be rewarded for their effort. The reward in most blockchain systems consists of a fixed block reward in addition to the fee for all transactions embedded in the block.

In this section, we first present background information about the Ethereum blockchain. Then, we provide an introduction to Gaussian Mixture Models that we used in this article to fit the data sets.

### Ethereum

Ethereum (Wood, 2019) is one of the most popular public blockchains that is designed to support customized decentralized applications. Ethereum enables users to write and run smart contracts on top of it with the assistance of its Turing-complete programming language. Smart contracts can be developed using high-level programming languages such as Solidity, and are executed within the Ethereum Virtual Machine (EVM). The currency of Ethereum is called Ether, and it is used to reward honest miners for their efforts toward maintaining the blockchain ledger.

In this section, we discuss specific aspects of the Ethereum blockchain that are relevant to the work described in this article. These aspects include accounts, transactions, EVM, PoW algorithm, blocks, and incentive mechanism (Wood, 2019).

#### Ethereum account

There are two different kinds of accounts in Ethereum, which are externally owned accounts (EOAs) and contract accounts. Both kinds of accounts have a unique address and a balance. Contract accounts differ from EOAs in that they can also have code and storage associated with them. Accounts can communicate with each other through transactions. The sender and the recipient of transactions are considered as accounts (in particular EOAs).

#### Ethereum transactions

Transactions are one of the main important elements in blockchain systems as they are responsible for updating the state of the blockchain. Transactions are included in blocks and then attached to the ledger. In Ethereum, there are three types of transactions, which are transfer, contract-creation, and contract-execution. Transfer transactions are to move Ether from one account to another. A contract-creation transaction is to deploy a fresh smart contract to the blockchain. Once the contract is deployed, it will be assigned to a specific address. Then, one could simply invoke that contract by sending a contract-execution transaction to the contract address to execute a particular function within the contract.

Transactions in Ethereum have different attributes. We focus on gas-related attributes (Gas Limit, Used Gas, and Gas Price) since they are relevant to our work in this article. Gas Price refers to the amount of Ether the submitter of the transaction is happy to pay per gas unit. Gas Limit refers to the maximum amount of gas units that can be consumed by the transaction, and it can be set by the sender of the transaction. The Gas Limit is to ensure transaction termination. Used Gas refers to the actual amount of gas units used by the transaction after executing it. The Used Gas for transfer transactions is fixed at 21,000 units of gas. For contract-related transactions, it depends on the complexity of the transactions. The more computational effort and storage space required the more Used Gas is needed. After executing and including a transaction in a block, the submitter of the transaction is charged the following fee: Used Gas $\times$ Gas Price. This is to compensate miners for their effort spent.

#### Ethereum virtual machine

The EVM is responsible for handling the execution of smart contract transactions (Wood, 2019). Every node in the blockchain network has a copy of the EVM to use for executing contract-creation and contract-execution transactions. The EVM supports various instructions (operation codes) such as PUSH and ADD, each of which has an associated cost. The cost of instructions is measured in a unit called Gas that we will discuss later under the incentive mechanism.

#### Consensus algorithms

Various consensus algorithms have been proposed for blockchain systems, including but not limited to, Proof-of-Work (PoW) and Proof-of-Stake (PoS). These algorithms are designed to maintain the blockchain ledger by reaching an agreement among the network nodes even with the presence of malicious nodes. In short, the PoW algorithm requires the network miners to invest their computing resources to participate in the consensus process, while miners are required to invest their coins in PoS to do so.

Here, we discuss the PoW algorithm since it is the current consensus mechanism used by the Ethereum blockchain. The PoW algorithm requires the network nodes to invest their computing resources to create and append new blocks to the blockchain ledger. The computing resources determine how often a miner can create new blocks to be added to the blockchain ledger. To create a new block, a miner has to find the right random number (called a nonce) that when combined with the block information results in a value with several leading zeros, and it is called the hash of the block. That is, miners have to repeatedly try different nonces until they find the correct block hash value, which makes this algorithm consume lots of energy. In Ethereum, the PoW algorithm is augmented with the longest-chain rule to resolve conflicts that may arise because of network propagation delays. We note that Ethereum is planning to move to more energy-efficient algorithms such as Proof of Stake (PoS). Ethereum slightly differs from Bitcoin in that it enables referencing stale blocks (*i.e*., blocks that are discarded after resolving the conflicts) in forthcoming blocks and rewarding their creators. Such stale blocks after being added to a future block are called uncle blocks. Transactions included in uncle blocks are discarded, and thus they do not contribute to the state of the blockchain.

#### Ethereum blockchain

The Ethereum blockchain is composed of a list of linked blocks. Each block has a header as well as contains a list of transactions and a maximum of two uncle blocks. The block header includes data relevant to the block such as block Gas Limit, block Used Gas, and other information. The block Gas Limit refers to the maximum amount of gas units that can be used by all transactions included in the block. The idea of the block Gas Limit is to limit the computation required by the network’s nodes and thus prevent denial of service attacks. It is essential as Ethereum supports complex smart contracts that may consume a large number of gas units. The block Used Gas indicates the total gas units used by all transactions included in the block. That is, a block can be considered as full if and only if the block Used Gas = block Gas Limit.

#### Ethereum incentive model

Ethereum has an integrated incentive model to provide incentives for miners who maintain and expand the blockchain ledger. The Ethereum incentive model rewards miners for creating and attaching new blocks to the ledger. The miner of a newly created block would receive a block reward, fees associated with transactions included in the block as well as rewards for every uncle block referenced. The block reward is a fixed amount of money (currently 2 Ether). The uncle block rewards are a small amount of reward for creating and referencing a stale block (Wood, 2019).

At the core of the Ethereum incentive model is the *Gas mechanism* that is purposely designed to limit the possible computation for smart contract transactions. This Gas mechanism is essential to avoid denial of service attacks as Ethereum enables writing complex smart contracts through its Turning-complete programming language. The purpose of the Gas mechanism is not only to limit the computation but also to provide fair rewards for miners for their effort spent.

Every instruction of a smart contract is assigned to a specific gas cost (Wood, 2019). The gas cost for instruction is set by the Ethereum foundation according to the computation and storage overhead required to execute that instruction on the EVM. For example, the cost of DIV instruction is five units of gas. When executing a contract transaction, the EVM counts the amount of Used Gas based on the instructions used by the transaction. The sender of the transaction is then charged for the amount of Used Gas, as discussed earlier.

It is essential to assure the fairness of the Ethereum incentive model to encourage miners to maintain the blockchain ledger honestly. Miners are often rational in that they may do whatever is permitted to maximize their revenue. One issue of the Ethereum incentive model is that miners receive a fixed amount of Ether as a block reward for every block created, regardless of how many transactions they include in their blocks. That is, miners might not fill up their blocks with transactions, and thus the system performance is negatively impacted, as we will discuss in “Proposed Dynamic Block Reward Approach”.

### Gaussian mixture models

This section provides an introduction to Gaussian Mixture Models (GMMs) and their application. Mixture models can accurately represent the characteristics of the given data sets (Huang, Peng & Zhang, 2017; Lindsay, 1995; Nguyen & McLachlan, 2019). This is due to their flexibility which makes them appropriate for modeling complex probability distribution functions (Zhuang et al., 1996). There are different types of mixture models such as Gaussian, and Categorical. The most common mixture model in the literature is Gaussian Mixture Models (GMMs) (Zhuang et al., 1996). In GMMs, multiple different normal distributions are fitted to the data to capture its shape accurately. This makes GMMs an appropriate choice when the shape of the data resembles multiple normal distributions. A mixture model $p(x|\Theta )$ can be defined as *K* weighted components,

(1) $$p(x|\Theta ) = \sum\limits_{i = 1}^K \; {\phi _i}\;p(x|{\theta _i}),$$

where, $x$ is the data sample to be fitted, $p(x|{\theta _i})$ is the $i$-th component. The parameters and the weight of the $i$-th component are defined as ${\theta _i}$ and ${\phi _i}$, respectively. The weight value of a component must be greater than zero ( ${\phi _i} \ge$ 0) and the mixture model must have a total weight of one for all the components ( $\sum\nolimits_{i = 1}^K {{\phi _i}} = 1$). Every component of the GMMs represents a normal distribution with the parameter ${\theta _i} = \{ {\mu _i},\sigma _i^2\}$, where the mean value is ${\mu _i}$ and the variance value is $\sigma _i^2$.

Before applying and fitting GMMs to the data set, two important parameters need to be identified, namely, the number of Gaussian components (*K*) and the appropriate parameter values for every component ( ${\theta _i}$) (Zhuang et al., 1996). Concerning the number of components, it should be carefully selected to protect against over-fitting and under-fitting (Huang, Peng & Zhang, 2017). Various methods have been proposed in the literature to help choose the right number of Gaussian components. Among the proposed methods, Akaike Information Criterion (AIC) and Bayesian Information Criterion (BIC) are the most widely used methods (Scrucca et al., 2016). The number of components that has the lowest value according to the AIC and BIC methods is the one that best fits the data. Concerning the parameters of the Gaussian components, different algorithms can be used such as Expectation-Maximisation (EM) (Dempster, Laird & Rubin, 1977). The way the EM algorithm works is that it relies on maximum likelihood estimation techniques to find the most appropriate parameters for every component.

In “Fitting Distribution to Data” we discuss how we implemented and applied Gaussian mixture models to obtain transaction distributions concerning Used Gas.

## Proposed dynamic block reward approach

In Ethereum, miners are responsible for maintaining and expanding the blockchain ledger by creating and appending new blocks. To create a new block, miners select and execute some pending transactions to be included in their block. In Ethereum, it is up to the miners to decide whether to fill up their blocks with transactions or not. This means that miners may generate blocks that only contain a few numbers of transactions or even do not contain any transactions. The issue is that producing non-full and empty blocks can significantly impact the throughput (the number of transactions executed per second) of the system.

The current incentive mechanism of Ethereum encourages miners to fill their blocks with transactions by collecting the fee for all included transactions. However, filling a block with transactions may not be preferred by miners for the following reasons. Firstly and most importantly, the largest proportion of the received incentive in Ethereum comes from the block reward. This reward is currently fixed at 2 Ether, and provided to miners for every block created regardless of the fullness of the block. As the block reward is independent of the included transactions, miners may not be well motivated to fill up their blocks with transactions. Secondly, it delays miners in the mining competition as they have to execute and validate more transactions before starting the mining process. Thirdly, it incurs a high network propagation delay and as a result, the block could lose the mining competition. To that end, producing non-full or empty blocks would result in high rewards as well as reduces the computation and communication overhead that may arise because of the included transactions.

To that end, it is crucial to design a proper incentive model that can assure that all blocks in the network are filled up with transactions. The incentive model should not leave any advantage for miners to create non-full blocks. To the best of our knowledge, no work in the literature investigates the problem of generating non-full blocks and their impact on the throughput of blockchain systems.

In this article, we conduct an extensive analysis to study this problem as well as propose a dynamic block reward approach in which the amount of block reward miners can get depends on how full is the block they created. The main motivation behind this approach is to leave very little advantage for miners to generate non-full blocks. Using our approach, miners would be motivated to fill up their blocks with transactions.

The proposed approach is easy to implement and integrate into Ethereum as we only need to change the block reward parameter. In our proposed approach, the block reward will be calculated based on the block utilization level, instead of being fixed to a particular amount. A block can have a utilization level of 100% if no more transactions can fit into the block. In particular, this can occur in Ethereum when the block Used Gas is equal to the block Gas Limit (Used Gas = Gas Limit). We can calculate the dynamic block reward ( ${B_{\rm DR}}$) as follows:

(2) $${B_{\rm DR}} = {B_{\rm UT}} * {B_\rm R}$$

where B_R_ is the maximum block reward miners would gain for every block created. This parameter can be set to 2 Ether as currently in Ethereum or any other value of interest. B_UT_ refers to the block utilization level (*i.e*., representing how full is the block). It can be calculated directly from the block information as follows:

(3) $${B_{\rm UT}} = \displaystyle{{{B_{\rm UG}}} \over {{B_{\rm GL}}}}$$

where ${B_{\rm UG}}$ indicates the block Used Gas and ${B_{\rm GL}}$ represents the block Gas Limit. The block utilization can only reach 100% if the block is full of transactions. In this case, the value of ${B_{\rm UG}}$ will be equal to that of ${B_{\rm GL}}$.

To demonstrate the usefulness of the proposed approach, let us assume there is a miner who only creates empty blocks. In the current Ethereum incentive model, the miner would receive 2 Ether for every block created. By applying our approach, the miner would receive nothing since the block utilization level is zero (block Used Gas = 0). From this example, we can see that the mining reward is proportional to the block utilization level. We will show more results and insights into our proposed approach in “Results”.

## Data collection and distribution fitting

To obtain insights into the impact of block utilization on the performance of Ethereum, we will parameterize the simulation with data collected from the Ethereum blockchain. We managed to collect the data for 500,000 Ethereum blocks that were randomly selected to avoid sample bias, covering the time duration from the launch of the Ethereum network until March 2022. This data embraces Gas Limit, Used Gas, and the number of transactions included in the block.

Table 1 show basic statistics about the collected data, including the minimum (min), the maximum (max), the mean, and the median for the collected block data. Block Gas Limit and Used Gas are given in million (M) units of gas. From the collected data, we calculated the block utilization and the average Used Gas for transactions, as we discuss in the following section. To be of use to the simulation in “Simulator and Validation of Simulation Results”, we fit the appropriate probability distributions to the block utilization and the transaction Used Gas.

**Table 1:** Statistics about the collected block data.

Data	Min	Max	Mean	Median
Block gas limit	3.9 M	30 M	13.8 M	8 M
Block used gas	0	30 M	8.6 M	8 M
Number of included transactions	0	1,428	121	107

### Design of data collection approach

To be able to analyze the impact of non-full blocks on the performance of the system, we need to observe block utilization from the Ethereum network. Using data-driven analysis can help us derive realistic and representative simulation results.

In this section, we propose an automated data collection approach to collect the details of Ethereum blocks. We managed to collect the data for 500,000 Ethereum blocks. From the collected data, we derive two important parameters that we will feed into the simulation (see “BlockSim Simulator Extension”), which are block utilization and transaction Used Gas.

#### Block utilization ( ${B_{UT}}$)

This parameter represents how full the block is. It can be calculated directly from the block information (see Eq. (3)).

#### Transaction Used Gas ( ${T_{UG}}$)

This parameter represents the size of the transaction. In Ethereum, the Used Gas of transactions can vary depending on the type and the data associated with the transaction. That is, the number of transactions that can fit in a single block differs from one block to another, and thus, the throughput of the system can be impacted. We calculated the average transaction Used Gas from the data we collected from the Ethereum network as follows:

(4) $${T_{\rm{UG}}} = \displaystyle{{{B_{\rm {UG}}}} \over {{B_{\rm{TX}}}}}$$

where ${B_{\rm TX}}$ denotes the number of transactions included in the block. Assume that the ${B_{\rm UG}} = 20,000,000$ and ${B_{\rm TX}} = 260$, then ${T_{\rm UG}} = 76,923$ units of gas.

### Fitting distribution to data

To utilize the collected data in our simulation, we need to fit distributions for two parameters, which are block utilization and transaction Used Gas.

For the transaction Used Gas values, we first attempted to fit general probabilistic distributions using the Fitter Python class (https://fitter.readthedocs.io/en/latest/). This class test 80 different distributions (*i.e*., normal, exponential, and gamma distributions) to the data and then plots the results of the most appropriate distributions and their best parameters. However, non of these simple probabilistic distributions fit the data particularly well as the data set is somehow complex. That is, we decided to select Gaussian Mixture Models (GMMs) as they are suitable for fitting distributions to complex data sets. We apply GMMs to the log of the data since it constitutes multiple normal distributions.

For the block utilization, we also attempted to fit general probabilistic distributions using the Fitter Python class, but we have not found any distribution that works with the data. To that end, we decided to fit a straightforward frequency distribution as the data values significantly vary. As the block utilization value ranges from 0% to 100%, we classified the frequency into seven categories as presented in Table 2. For each category, we show the number of observed blocks (out of 500,000 collected blocks) and their contribution percentage. The first category, for instance, represents the case of empty blocks (0% block utilization) that do not contain any transactions. This category accounts for 6.57% of the total collected blocks as there are about 33,000 empty blocks out of 500,000 blocks. The last category where the block utilization ranges from 95% to 100% represents the case when the block is almost full of transactions.

**Table 2:** Frequency distributions of Ethereum block utilization.

Block utilization	Number of blocks	Percentage
0%	32,871	6.57%
>0% and $\le$20%	102,550	20.5%
>20% and $\le$40%	38,431	7.68%
>40% and $\le$60%	27,970	5.59%
>60% and $\le$80%	23,176	4.63%
>80% and $\le$95%	19,715	3.94%
>95% and $\le$100%	255,538	51.08%

**Figure 1:**
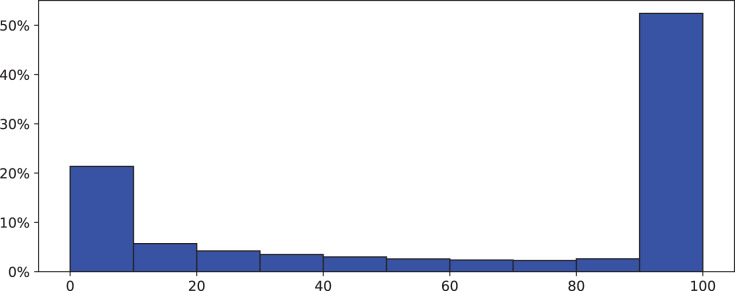
Frequency distribution of Ethereum block utilization.

From Table 2 and Fig. 1, we can observe that only 51% of the blocks collected from the Ethereum network are almost fully utilized. About a quarter of the collected blocks has a utilization level of less than 20%, and of that 6.5% is empty blocks.

In addition, we are interested in figuring out whether block utilization levels generally change over time or not. To achieve this, we inspect the creation time and the utilization level for every block in the collected data set. We have not found a correlation between the utilization level and the creation time of Ethereum blocks. That is, the distribution for the block utilization level is not time-invariant. It might be impacted by the behavior of the participating nodes since nodes are not enforced to fill their blocks with transactions as we stated in “Proposed Dynamic Block Reward Approach”.

Algorithm 1 presents the procedure for fitting distributions to the parameters and that for sampling from such distributions. To fit a GMM to the log Used Gas for transactions, we have to estimate the number of Gaussian components *K*. For each component, we have to estimate its mean ${\mu _i}$, variance $\sigma _i^2$, and weight ${\phi _i}$. To determine *K*, we apply two of the most widely adopted methods, namely the Bayesian Information Criterion (BIC) and Akaike Information Criterion (AIC) (Scrucca et al., 2016). We evaluated different values of *K* and then selected the best *K* value based on these criteria. To determine the mean, variance, and weight for each component, we apply the Expectation-Maximisation (EM) algorithm (Dempster, Laird & Rubin, 1977). After estimating the appropriate parameters, we fit the GMM to the data and then we sample from the fitted distribution.

**Algorithm 1:** The fitting and sampling procedure

**Procedure** *Fit a GMM to log* ${T_{UG}}$
Determine *K* ▻Use AIC/BIC
Estimate $\sum\nolimits_{i = 1}^K {{\mu _i}} ,\sum\nolimits_{i = 1}^K {\sigma _i^2,\sum\nolimits_{i = 1}^K {{\phi _i}} }$ ▻Use EM algorithm
$P \leftarrow GMM(K,\sum\nolimits_{i = 1}^K {{\mu _i},\sum\nolimits_{i = 1}^K {\sigma _i^2,\sum\nolimits_{i = 1}^K {{\phi _i}).} } } \,\rm{fit}(\log ({T_{UG}}))$
${T_{UG}} \leftarrow \exp (P.sample(n))$ ▻Sample Transaction Used Gas
**EndProcedure**
**Procedure** *Fit a frequency distribution to* ${B_{UT}}$
$rand \leftarrow Unif(low = 0,high = 100,size = n)$
▻Sample Block Utilization
** if** $rand < 6.57$ **then**
${B_{UT}} \leftarrow 0$
** else if** $rand < 27.07$ **then**
** ${B_{UT}} \leftarrow Unif(0,20)$**
** else if** $rand < 34.75$ **then**
** ${B_{UT}} \leftarrow Unif(20,40)$**
**else if $rand < 40.34$ then**
** ${B_{UT}} \leftarrow Unif(40,60)$**
** else if** $rand < 44.97$ **then**
** ${B_{UT}} \leftarrow Unif(60,80)$**
** else if** $rand < 48.91$ **then**
** ${B_{UT}} \leftarrow Unif(80,95)$**
** else**
** ${B_{UT}} \leftarrow Unif(95,100)$**
** end**
**EndProcedure**

To fit a frequency distribution to the block utilization, we used uniform distributions to draw a random number (*rand*) that ranges from 0 to 100. Then, we sample a block utilization value ( ${B_{UT}}$) from a uniform distribution based on the value of *rand*. For example, if *rand* is less than 6.58, then ${B_{UT}}$ will be zero. This is to capture the frequency distributions stated in Table 2.

We implemented our algorithmic procedure in Python using the machine learning library Scikit-learn since it includes the GaussianMixture package. The EM algorithm and the AIC/BIC criteria are all implemented in the GaussianMixture package. We implemented a Python class to fit distributions to the desired parameters as well as to sample values from the fitted distributions.

**Figure 2:**
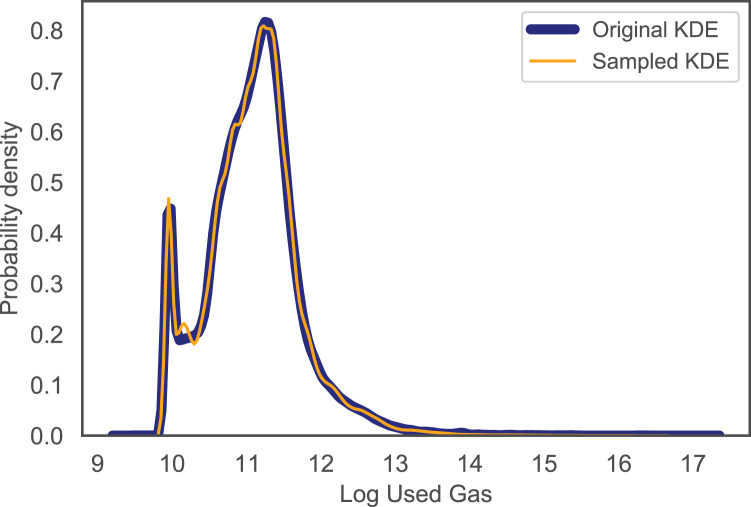
KDE for original and sampled used gas.

**Figure 3:**
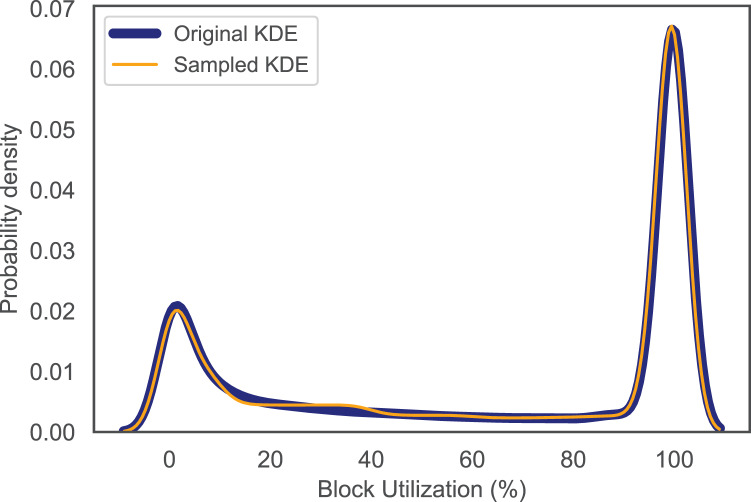
KDE for original and sampled block utilization.

We evaluated the accuracy of the fitted distributions by comparing the Kernel Density Estimation (KDE) for the original Used Gas and block utilization data with that of the sampled ones. This is to assess how close the data samples are to the original data. From Figs. 2 and 3, it is obvious that the KDE for the sampled data matches that of the original one for both Used Gas and block utilization. That is, our fitting approach is capable of capturing the shape of the actual data.

## Simulator and validation of simulation results

To conduct our model-based approach to analyzing the impact of non-full blocks on the system performance, we used and extended the publicly available BlockSim simulator. In this section, we first demonstrate how we extended the simulator and then we validate our simulation results with that of the real Ethereum system.

### BlockSim simulator extension

BlockSim (Alharby & van Moorsel, 2019, 2020) is a generic discrete-event simulator for blockchain systems. It provides extensible simulation constructs that can be used by blockchain researchers to investigate various non-functional properties such as performance indicators (throughput and latency) and system properties (*e.g*., mining fairness and incentives). BlockSim is structured in three different layers, which are consensus, incentives, and network. At the core of BlockSim is a base model that is designed to cover the main elements common across the majority of blockchain systems. These elements include blocks, transactions, nodes, consensus resolution, and incentive distribution. This base model can be extended to simulate any blockchain system of interest. It has been extended to include both Bitcoin and Ethereum models. The BlockSim simulator has been validated against existing blockchain implementations and studies from the literature, and it is publicly available.

To support our analysis in this work, we introduced the following modifications:

#### The attributes of blocks

We extended the Block class to include an additional attribute required by the model, which is the block utilization. This attribute represents how full is the block. Thus, each block created in our simulations has this attribute.

#### The distribution fitting class (Fitter)

We introduced a new class named Fitter to fit probability distributions to the block utilization and transaction Used Gas parameters. This follows the procedure we explained in “Fitting Distribution to Data”. We execute the fitting method once at the start of the simulator. Then, when creating new blocks and transactions, we sample random values for these parameters from the fitted distributions.

#### The strategy of mining

The BlockSim simulator relies on the assumption that miners always fill their blocks with transactions. To simulate the case where some blocks are not full or even do not contain any transactions, we extend the Node class to configure mining strategies. For example, one could set a miner to generate blocks of any utilization level.

#### The incentive mechanism

To assess the usefulness of the proposed dynamic block reward approach, we modify the Incentive class of the BlockSim simulator. In particular, we introduce a dynamic block reward based on the value of the block utilization, instead of being fixed to a particular amount.

### Validation of simulation results

In this study, we will use the BlockSim simulator after feeding it with the distributions that we derive from the real Ethereum data. In this section, we validate our simulation results with that of the real Ethereum network to ensure that we can obtain realistic and representative simulation results.

We use the following relevant metrics for validation: the number of blocks generated per day (B_day_), and the number of transactions completed per second (throughput). Table 3 compares the simulation results with that from the actual Ethereum system. We report both the average and the 95% confidence interval values, for a run of the simulation that corresponds to a full month of real-time.

**Table 3:** Validation of the simulator results by comparison with measurements from Ethereum. B_day_ is the total number of blocks appended to the blockchain ledger per day, and throughput is the number of transactions processed per second.

Metric	Measured results	Simulated results
B_day_	$6,\!074 \pm 27$	$6,\!078 \pm 25$
Throughput	$13.95 \pm 0.18$	$14.09 \pm 0.20$

From Table 3, we can see that our simulation results are close to that of the real system. That means the data we collected from the Ethereum network is representative and the distributions we fit the data are appropriate.

In the following section, we will compare the performance results of the current Ethereum with the case when miners always fill their blocks. In addition, we will compare the mining incentive results of the current Ethereum incentive mechanism with that of our proposed dynamic block reward mechanism. Our analysis will cover both the current and future implementation of Ethereum.

## Results

In this section, we present the main findings regarding the impact of block utilization on the throughput of the Ethereum network. In addition, we show the findings that support the effectiveness of the proposed approach for dynamic block rewards. Our main metrics of interest are throughput and mining incentives. The throughput indicates the number of transactions that can be processed per second, while mining incentives indicate the amount of reward (in Ether) miners would receive.

We summarize the main findings that follow from our discussion upfront:

- The throughput of the Ethereum system is currently far from its optimal level as a large number of blocks is not fully utilized. The throughput can be improved by 56% if all blocks are full with transactions.
- Although increasing the block limit can result in higher throughput, there is no assurance that the throughput will reach the optimal level as this depends on the behavior of miners.
- In the current Ethereum incentive mechanism miners of non-full or empty blocks can still receive some rewards, although it is less than that of full blocks.
- The proposed dynamic block reward approach leaves no advantages for miners who create empty blocks. Miners of empty blocks will always receive nothing.
- The proposed approach significantly punishes miners who do not fill up their blocks with transactions. For instance, when miners only fill up to a fifth of their blocks, they can receive 80% less reward than what they are receiving in the current incentive mechanism of Ethereum. That is, our proposed approach may result in enforcing and pushing miners to produce full blocks.

### Throughput results

From the data we collected from the Ethereum network (see “Data Collection and Distribution Fitting”) we found that almost half of the generated blocks are not well occupied with transactions. Even worse is that we found about 6% of the total blocks are empty. This indicates that the throughput of the Ethereum system is far from its best and expected performance level.

The main motivation of this section is to demonstrate to which level the performance of the Ethereum system can be improved when miners utilize their blocks properly as expected (*i.e*. filling blocks with transactions). To achieve this, we run different simulations that capture the current situation in Ethereum where miners do not always fill their blocks with transactions. We also capture the best scenario where miners always produce full blocks. Comparing the two scenarios can give us insights into the current performance of Ethereum as well as the best achievable level. We note that we consider the current and future implementation of Ethereum in terms of block limits (in million (M) units of gas) to see how it can affect the performance of the system. We simulate each configuration setting for 1 day of the real Ethereum network time, and then we report the average result from 30 independent runs. The confidence interval is not stated here, but it is within 1.5% of the average value.

**Figure 4:**
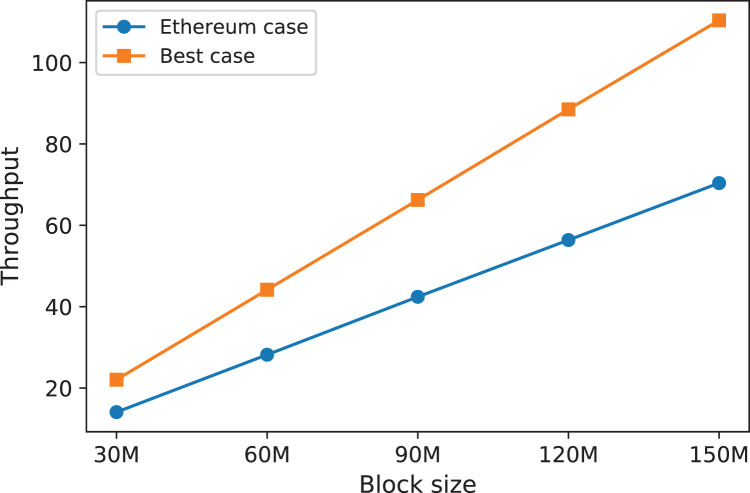
The throughput for both the Ethereum case and the best possible case, for different block limits.

Figure 4 shows the throughput, for different block limits. The blue curve represents the throughput as in the current case of Ethereum where miners do not fully utilize their blocks. That is, one would expect to see non-full or empty blocks. The orange curve represents the optimal throughput level where miners always fill their blocks with transactions.

From Fig. 4 we conclude that for the current implementation of Ethereum (block limit = 30M and block utilization is the same as presented in “Data Collection and Distribution Fitting”), the throughput is 14 transactions per second. If all miners, however, fill their blocks with transactions, the throughput will be pushed to 22 transactions. In other words, producing full blocks would result in improving the throughput of the current Ethereum by about 56%. That means about 700,000 additional transactions can be processed per day.

We can also see from Fig. 4 that increasing the block limit would result in higher throughput. For instance, increasing the block limit from 30 M to 60 M would double the throughput level. This somehow is expected as it means more transactions can fit into the block. Over the past years, the block limit of Ethereum has been increased from about 2M in 2015 to 30 M at the time of writing this article. The reason for such an increase is to improve the throughput of the system. However, from Fig. 4 we can see that increasing the block limit can improve the throughput to a certain degree, the optimal level is not guaranteed as it depends on the behavior of miners who created the blocks. For example, the throughput will remain the same when miners produce empty blocks regardless of how much the block limit was increased. That is, it is crucial to have an effective incentive mechanism that ensures all blocks are fully utilized to push the throughput to the optimal level. The incentive mechanism must leave no advantages for miners to produce non-full blocks. In the following section, we will shadow the light into this.

### Incentive results

As we discussed in “Proposed Dynamic Block Reward Approach”, the current Ethereum incentive mechanism neither enforces nor motivates miners well to fill their blocks with transactions. That is, the focus of this section will be on investigating the mining incentives within the current Ethereum incentive mechanism as well as within our proposed mechanism demonstrated in “Proposed Dynamic Block Reward Approach”.

We run different simulations, with different block limits. We consider seven different miners, each with a different strategy in terms of block utilization, as depicted in Table 4. For example, Miner *M1* always produces empty blocks, while miner *M7* generates blocks with a utilization level ranging from 95% to 100%. We set the hash power for all miners to 14.29%. This is to avoid the impact of the invested hash power on the gained reward. The block limit and the Gas Price for transactions are set to the current values of 30 million units of gas and 100 Gwei respectively. Each configuration setting represents a simulation of 1 day of the Ethereum network. The reported results here are the average value obtained from 30 independent runs.

**Table 4:** Configuration of miners in terms of hash power used and block utilization strategy.

Miners	Hash power	Block utilization
M1	14.29	0%
M2	14.29	>0% and $\le$20%
M3	14.29	>20% and $\le$40%
M4	14.29	>40% and $\le$60%
M5	14.29	>60% and $\le$80%
M6	14.29	>80% and $\le$95%
M7	14.29	>95% and $\le$100%

**Figure 5:**
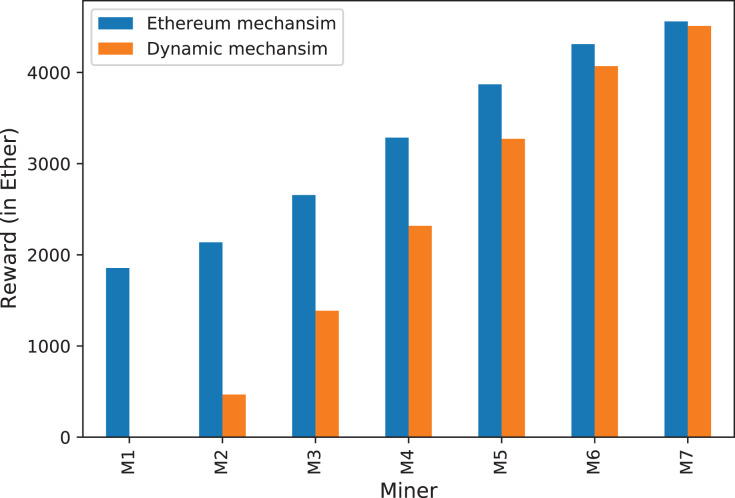
The mining incentives for miners (M1, M2,.., M7) within the Ethereum case and the proposed dynamic approach.

Figure 5 shows the mining rewards per day that miners would receive based on their block utilization strategies, for different block limits. The blue bars represent the rewards miners would get in the current Ethereum incentive mechanism, while the orange bars show that for our proposed dynamic approach.

From Fig. 5, we can observe the following main insights regarding the current Ethereum incentive mechanism. First, the higher level the of block utilization the more rewards miners would get. This is because miners would include more transactions in their blocks, and thus gain more rewards by collecting the fee for those additional transactions. However, the simulator does not take into consideration the time delay imposed on miners by executing and including transactions in their blocks. Thus, we might expect less reward for miners with higher block utilization levels when considering transaction processing overhead, as we will discuss in “Discussion”.

Second, miners who produce non-full or empty blocks can still gain some significant rewards. For example, miners who always generate empty blocks can collect about 1,855 Ether per day (almost 8% of the total rewards distributed in the network). Although such miners receive less reward than their invested hash power (14.29%) as they miss the transaction fees, they can benefit from the collected block rewards. The issue here is that empty blocks are useless in that they do not contribute to the network. Such empty blocks reduce the performance level of the system as shown in the previous section.

We believe that when miners still benefit from producing non-full or empty blocks they might continue to do so. This motivates us to propose an adjustment to the Ethereum incentive mechanism to discourage miners from generating non-full or empty blocks.

The idea behind the dynamic block reward approach (“Proposed Dynamic Block Reward Approach”) is to diminish the rewards miners would receive for non-full and empty blocks. This is to enforce miners to fill their blocks with transactions, thus improving the throughput of the system.

To assess the usefulness of this approach, we modify the Incentive class of the BlockSim simulator to calculate the block reward based on how full the block is. We replicate the results that we present earlier in this section but apply our purposed approach of dynamic block rewards. The results of our proposed dynamic approach are also depicted in Fig. 5 (the orange bars).

From Fig. 5 we can observe the following insights regarding our proposed approach. First, our approach of dynamic block reward does not leave any incentives for miners who generate empty blocks whereas in the current Ethereum miners can gain rewards for creating empty blocks. As we mentioned earlier, empty blocks do not contribute to the performance as they do not contain any transactions. Second, there is a clear advantage for miners who add more transactions to their blocks. for example, a miner who fills up to 20% of the block would only receive a third of the incentives assigned for miners who have a block utilization of 40%. This means that the mining rewards can be significantly reduced when the block utilization level is low. As a comparison to the current Ethereum incentive mechanism, our proposed approach is effective as it punishes miners who do not fill their blocks with transactions. For example, miners who only fill up to 20% can have their rewards reduced by about 80%.

To summarize, having a dynamic block reward that depends on the block utilization level could discourage miners from producing non-full or empty blocks. Miners would be motivated to include as many transactions as possible in their blocks to maximize their block rewards. As this approach does not provide incentives for generating empty blocks, one would not expect to see such blocks. As a result, the performance of the system would be improved as miners include more transactions in their blocks.

## Discussion

Our analysis in this work is not limited to the Ethereum network, but it can be applied to other blockchains with similar incentive mechanisms such as Bitcoin. That means our dynamic block reward can be useful to any blockchain system that relies on a fixed block reward. In this section, we first discuss the validity threats to our study. Then, we discuss the potential security issues and adoption challenges toward the application of our proposed approach.

### Validity threats

In this section, we discuss the major validity threats to our evaluation of the impact of non-full blocks on the system performance and mining incentives.

#### Transaction processing overhead

In “Incentive Results” we show the mining incentives for miners with different block utilization levels. It is worth noting that the BlockSim simulator does not account for the transaction processing overhead. Miners will be introduced to a time delay for processing and executing transactions in their blocks. This delay depends on the block limit and the block utilization level. The larger or the full the block the more delay will be introduced. For example, it could on average take 3.18 s to fill a block of 128 million units of gas (Alharby et al., 2020). This time delay might delay miners in the mining competition, and thus, the rewards miners would get from utilizing their blocks might be less than what we show in this article. That might be the reason why miners are still doing empty blocks and scarifying the transaction fees. However, we believe that the results of this study are still valid as miners receive significant rewards for non-full blocks.

#### Reduction of the block reward

The block reward is decreasing over time and might be removed eventually (Hu et al., 2019). This would leave transaction fees as the only incentive for miners. Without block rewards, miners will be well motivated to include as many transactions in their blocks as possible to maximize their revenue. This means that the issue we raised in this study might not be as significant as it is now. However, our analysis is still valid for the following reasons. First, miners might generate non-full blocks to avoid the overhead of processing transactions. Second, the block reward is not expected to be removed soon. For example, it is expected to be removed from the Bitcoin network by 2140. The issue we raised in this work exists as long as there is a block reward. Thirdly, the Ethereum improvement proposal (EIP-2878 (https://github.com/ethereum/EIPs/pull/2878)) for changing the Ethereum block reward from 2 to 0.5 Ether has received lots of criticism from miners. The block reward is essential to keep miners more interested in securing the blockchain network. Instead of removing the block reward, our proposed approach might be a better solution.

#### Different consensus algorithms

Our analysis in this study is based on the proof-of-Work (PoW) consensus protocol as currently used in Ethereum. However, Ethereum is planning to move to efficient protocols such as Proof-of-Stake (PoS) (Buterin & Griffith, 2017; Wang et al., 2019). Our analysis can stay even when shifting to PoS since the incentive mechanism will stay the same. There will be a block reward and miners would have the decision of filling up their block with transactions or not. To that end, the issue we raised will also be valid on different consensus algorithms.

### Adoption challenges and security considerations of the proposed approach

Our proposed dynamic block reward approach aims at promoting block utilization by encouraging miners to include as many transactions in their blocks as possible, which as a result, improves the performance and security of the blockchain system. In this section, we discuss how the proposed scheme can work and be integrated into the real Ethereum blockchain without imposing security issues or adoption challenges.

#### Adoption challenges

Our analysis of the proposed dynamic block reward scheme shows that the block creation incentives for miners can be reduced in case of generating non-full blocks. The principle of our proposed approach is to ensure the incentives received by miners are proportional to the block utilization level. That is, the amount of rewards miners would receive is not reduced unless they do not fill their blocks with transactions. Rational miners can still receive the same amount of incentives they receive before by filling their blocks with transactions.

To integrate our proposed scheme into the current Ethereum network, there is a need to update the existing software that runs on every network node. Such software updates can be either backward-compatible or non-backward-compatible. A backward-compatible software update (also called a soft fork) does not require every node to upgrade its software. Nodes can either follow the new or the old version of the software. The Bitcoin network’s SegWit upgrade is an example of a soft fork (Vujičić, Jagodić & Ranđić, 2018). On the contrary, a non-backward-compatible software update (also called a hard fork) requires every node to upgrade its software. The work conducted by a node that follows the old version of the software will not be accepted by other nodes following the new upgrade. This type of network fork often results in conflicts as some nodes might follow the new version of the software while others still work on the old version. An example of this type of fork is the Casper update in the Ethereum network to move from PoW protocol to PoS protocol (Buterin & Griffith, 2017). Any node that does not follow the Casper update will become incompatible with other nodes following the software upgrade.

In the case of our proposed scheme, a hard fork is required to ensure that all nodes on the network update their software. This is to assure that the proposed incentive mechanism is applied to everyone. If a soft fork is chosen, then nodes that follow the old version would receive the full block reward regardless of how full their blocks are. As hard forks often result in a split in the community, there might be some adoption challenges to be considered. We discuss some of these adoption challenges and highlight how our scheme can effectively work in practice despite these challenges.

The first challenge to consider when the Ethereum blockchain updates its software to accommodate our proposed scheme is that nodes may not follow that update, and instead, they would continue using the old version of the Ethereum software. As commonly known, the security of the blockchain network relies on the assumption that the majority of the nodes are honest in that they would follow the protocol and all forthcoming updates (Xiao et al., 2020). Regarding the proposed scheme, there might be some nodes that reject it, causing a blockchain fork. However, the proposed scheme would work in practice as long as most of the nodes are honest. When most of the nodes accepted the software update, rational or malicious nodes should also accept it, or otherwise, they will continue working on an abandoned blockchain ledger branch.

The second challenge is that even if nodes are enforced to follow the software update that includes our proposed scheme, they can create enough fabricated transactions to fill their blocks to avoid the potential reward reduction. It is hard (if not impossible) to prevent nodes from creating and including their own transactions in permissionless blockchain networks. This is especially true since nodes are pseudonymously identified in the network, and thus, it is feasible to find several nodes belonging to a particular node that can be used to populate the network with fabricated transactions. However, transactions in such blockchain networks have associated costs, which means that nodes have to pay a fee for every transaction executed and embraced in a block. That is, nodes might include transactions from the network to fill their blocks to maximize their revenue, instead of creating costly fabricated transactions. In addition, our proposed scheme focuses on improving the performance of blockchain systems by enforcing miners to include more transactions in their blocks. Therefore, it does not matter if nodes include their transactions or not as the overall performance in terms of throughput will stay the same.

The third challenge to raise is that nodes may have to wait for enough transactions before performing the mining process for the next block, resulting in losing the mining competition with other network nodes. However, we believe this is not the case as the arrival rate of Ethereum transactions is significantly more than what can currently be processed by the Ethereum network. According to Etherscan (https://etherscan.io/), the Ethereum network currently experiences between 1,000 to 2,500 new transactions per second. This number is expected to increase linearly with the growth of the Ethereum network in terms of the number of network nodes, smart contracts, and decentralized applications. We stated in “Results” that the current implementation of Ethereum can handle about 14 transactions per second and this number can rise to 22 when integrating our proposed scheme. These numbers are still far from the number of transactions entering the Ethereum network. That is, nodes may not have to wait for more transactions to arrive before starting the mining process of blocks.

One could still argue that even if the blockchain network experiences a significant number of new transactions compared to what can be tolerated, some of these transactions could be low-fee, conflicting, or malicious ones. However, even with the existence of conflicting, malicious, or low-fee transactions, we believe that nodes might not face a situation where they cannot fill their blocks because of this. This is especially true as the number of newly arrived transactions is expected to increase over time when more decentralized applications are built on top of the network. In addition, nodes can still choose which transactions to include in their blocks. For example, they can ignore low-fee transactions, especially when there are other high-fee transactions.

The last challenge that can be argued is that filling blocks with transactions may incur time delays in validating the block’s transactions. That is, nodes might prefer to not fill their blocks with transactions. However, the time delay required for validating transactions is by far less than the PoW delay. In addition, permissionless blockchains (including Ethereum) are considering the move to more energy-efficient consensus protocols such as PoS, which removes the need for performing heavy computation work and removes the mining competition among the network nodes. When shifting to PoS protocol, for instance, the impact of such time delay is even more negligible as the cost of running a network node will be significantly diminished.

#### Security considerations

Our proposed approach is an adjustment to the integrated incentive mechanism. It has no direct security issues since the core consensus mechanism is not altered. That is, common attacks such as Sybil and 51% attacks do not threaten our approach. The only concern that may arise when blocks are fully utilized with transactions is that it imposes computational effort on other nodes in the network as those nodes should check and verify every recipient block and its transactions. As a result, nodes might not verify the recipient blocks, making the security of the blockchain network under threat (Alharby et al., 2020; Luu et al., 2015). However, this security issue can also occur in the current implementation of Ethereum as various miners create full blocks with transactions. Also, the block verification process is by far less complex as opposed to the mining process, which makes this threat negligible.

## Related work

In this section, we discuss the current literature related to the performance evaluation of public blockchains such as Ethereum. In addition, we discuss how our work contributes to the state-of-the-art literature.

Croman et al. (2016) conduct an extensive analysis to understand the factors that may contribute to performance bottlenecks in the Bitcoin network. From their analysis, they found that the way to scale the network should be by increasing the block size or/and reducing the block generation rate. In Kawase & Kasahara (2017), the authors propose a queuing theory-based model to evaluate and analyze the latency of Bitcoin transactions. Gervais et al. (2016) evaluate the performance of PoW blockchains in terms of different network parameters such as block size, block generation rate as well as the underlying propagation mechanism. Yasaweerasinghelage, Staples & Weber (2017) analyze and predict transaction latency in blockchain systems using an architectural performance modeling approach. Papadis et al. (2018) evaluate how block propagation delay and mining hash power could result in more stale blocks, thus reducing the performance of the system. Their analysis makes use of stochastic models, and it is only applicable to PoW blockchains. Kim & Kim (2021) propose a method for reducing the block generation rate to improve the throughput of PoW blockchains. To improve the performance of permissionless blockchains, the authors in Hassanzadeh-Nazarabadi, Küpçü & Özkasap (2021) propose a blockchain architecture that utilizes Distributed Hash Table (DHT) of nodes for retrieving blocks and transactions on demand.

There is some effort to evaluate and analyze the performance of blockchain systems. However, the majority of related work focuses on the network and consensus parameters and their impact on the system performance. These parameters embrace block size, block generation rate, network propagation mechanism, and the hash power of the network nodes. To the best of our knowledge, there is no existing work that shadows the light on the impact of producing non-full blocks on the performance of the system. That is, our work aims to bridge this gap by providing an extensive data-driven analysis of this.

Some work in the literature focuses on the incentive mechanism of Ethereum, and its relation to the system performance. For instance, the authors in Aldweesh et al. (2021) propose OpBench as a performance benchmark system for Ethereum Virtual Machine. This system is to evaluate the alignment of CPU usage for smart contract instructions with the associated fee for these instructions. In Alharby & van Moorsel (2021), the authors analyze through simulation different strategies for transaction selection, and their impact on the received mining rewards. They found that miner could increase their revenue by discarding and including some types of transactions (*i.e*., based on the time needed to process transactions). When miners commit such behaviors the performance of the system would be impacted as some transactions may take a long time waiting to be confirmed in the blockchain ledger. In Alharby et al. (2020), the authors analyze the lack of verification incentives in Ethereum and its impacts on the correctness of the blockchain data. To address this issue, they proposed a parallel verification approach that speeds up the verification process, thus improving the system performance.

Our work differs from the above-mentioned studies in that we provide, to the best of our knowledge, the first extensive analysis to study mining incentives under different configurations of block utilization. This is to understand the behavior of miners who generate non-full blocks. For example, how much reward miners would receive if they created full, non-full, or empty blocks, as we do in this work. The incentive mechanism, if it is properly designed, can improve the system performance by encouraging miners to fill their blocks with transactions to reach the optimal performance level.

## Conclusion

This article provides the first extensive data-driven analysis of the impact of non-full blocks on the performance of the Ethereum blockchain, using discrete-event simulation techniques. To draw realistic simulation results, we collected the necessary data from the Ethereum network and then transform it into distributions to parameterize the simulator. The main insight we gained from our analysis is that the performance of Ethereum is far from its optimal level, despite the efforts spent over the years through increasing the block limit to handle more transactions. We show that the performance level could be improved by at least 50% if all blocks in the network are fully utilized with transactions. Our results suggest that the poor performance level of Ethereum is due to the behavior of miners as well as the design of the current incentive mechanism. That is, we proposed a dynamic incentive mechanism that rewards miners based on how full their blocks are, which in turn leaves very little advantage for miners to produce non-full blocks. This is to enforce miners to fill their blocks with transactions, and thus the system performance will reach its desirable level. Our scheme can be securely applied to the Ethereum blockchain.

## Additional Information and Declarations

### Data Availability

The following information was supplied regarding data availability: Data, scripts, and simulators are available at Figshare: Alharby, Maher (2022): PeerJ row-data and code.zip. figshare. Dataset. https://doi.org/10.6084/m9.figshare.21114244.v1.
